# Preparation and Applications of Green Thermoplastic and Thermosetting Nanocomposites Based on Nanolignin

**DOI:** 10.3390/polym14245470

**Published:** 2022-12-14

**Authors:** Debora Puglia, Francesca Luzi, Luigi Torre

**Affiliations:** 1 Department of Civil and Environmental Engineering, University of Perugia, 05100 Terni, Italy; 2 Department of Materials, Environmental Sciences and Urban Planning (SIMAU), Polytechnic University of Marche, 60131 Ancona, Italy

**Keywords:** bio-based, thermosetting, thermoplastic, nanocomposites, lignin nanoparticles

## Abstract

The development of bio-based materials is of great importance in the present environmental circumstances; hence, research has greatly advanced in the valorization of lignin from lignocellulosic wastes. Lignin is a natural polymer with a crosslinked structure, valuable antiradical activity, unique thermal- and UV-absorption properties, and biodegradability, which justify its use in several prospective and useful application sectors. The active functionalities of lignin promote its use as a valuable material to be adopted in the composite and nanocomposites arenas, being useful and suitable for consideration both for the synthesis of matrices and as a nanofiller. The aim of this review is to summarize, after a brief introduction on the need for alternative green solutions to petroleum-based plastics, the synthesis methods for bio-based and/or biodegradable thermoplastic and thermosetting nanocomposites, along with the application of lignin nanoparticles in all green polymeric matrices, thus generating responsiveness towards the sustainable use of this valuable product in the environment.

## 1. Introduction

The rising global demand for fossil resources for nonenergy purposes, as in the case of plastics production, has intensely motivated research to find alternative solutions to petrochemical plastics; however, progress has still not reached a commercially viable scale. The demand for cost-effective, ecofriendly materials has also increased to reduce waste management and pollution issues, thus academic/industry interest in sustainable bio-based materials has accordingly exploded in recent years. Even if numerous synthetic biopolymers have been used to this purpose, the need to suit different applications has allowed for progress even in the field of natural biopolymers. According to this, significant progress in lignin valorization and the use of sustainable and natural resources has been accomplished in the last years, in particular with regard to bio-based and biodegradable natural polymers based on this source, which are facing increasing consideration as they are environmentally green and economically reasonable. Replacing petroleum-based-derived materials with sustainable and environmentally friendly materials has been also considered as a crucial activity in the current period, so consideration has been given to the progress of lignin bio-based and/or biodegradable materials with thermomechanical performance that can compete or even surpass the petroleum-based products presently used. Lignin is considered an excellent substitute feedstock for the preparation of chemical products and polymers, even if one of the main difficulties still remaining is the lack of a well-defined structure and the partial flexibility linked to its origin, including extraction fragmentation procedures [1]. Although lignin is presently often considered as a filler or additive, it is hardly appreciated as a natural resource for chemical production. Nevertheless, it may be an outstanding candidate for chemical reactions due to its extraordinary reactivity (i.e., the presence of abundant aliphatic and phenolic hydroxyl groups) for the preparation of bio-based materials.

Following its fast development, efforts have incessantly been made to advance its compatibility with other additives in multicomponent systems, as in the case of its mixing with thermoplastics and thermosetting green matrices [2]. On the other hand, in parallel to its application at the macroscale as an additive in bio-based polymeric materials, research has advanced to solve these limits and has opened a different perspective towards the use of lignin-based nanomaterials as functional fillers in bio-based matrices [3]. To realize the potential of this material, one of the possible routes to follow includes lignin use in nanocomposite assemblies, where synergic interactions are extremely advantageous [4]. To this end, we review both existing possibilities, i.e., renewable thermoset and thermoplastic polymers based on lignin and the use of nanolignin as the active ingredient in these specific matrices, with particular attention to their application in niche sectors.

## 2. Synthesis of Bio-Based Polymeric Matrices from Lignin

**Figure 1:**
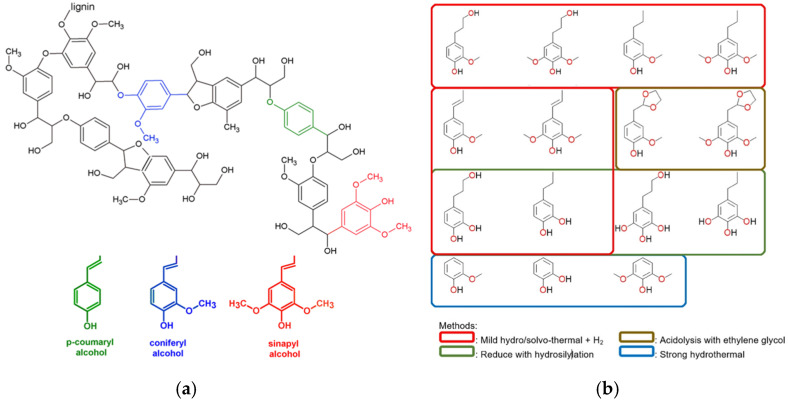
(**a**) Typical fragments of lignin structure with its main monolignols and (**b**) the potential phenolic products from lignin degradation. Reprinted with permission from Refs. [8,9]. Copyright 2019, Springer Nature Switzerland AG & Wiley.

Lignin is characterized by a complicated three-dimensional structure, obtained by polymerizing three phenylpropane units that arise from three aromatic alcohols: p-coumaryl alcohol, coniferyl alcohol, and sinapyl alcohol (Figure 1a) [5].

Its complexity can be controlled by accurate deconstruction, so the synthesis of polymers starting from aromatic compounds ideally obtained from lignin has attracted a growing curiosity. A large quantity of polymers obtained from lignin derivatives originate from different products (Figure 1b), gained by applying various depolymerization techniques [6,7]: as a function of the phenol substrates, various chemical changes and polymerization routes can be settled, leading to (semi)aromatic polymeric systems covering a widespread range of diverse thermal and mechanical characteristics.

**Figure 2:**
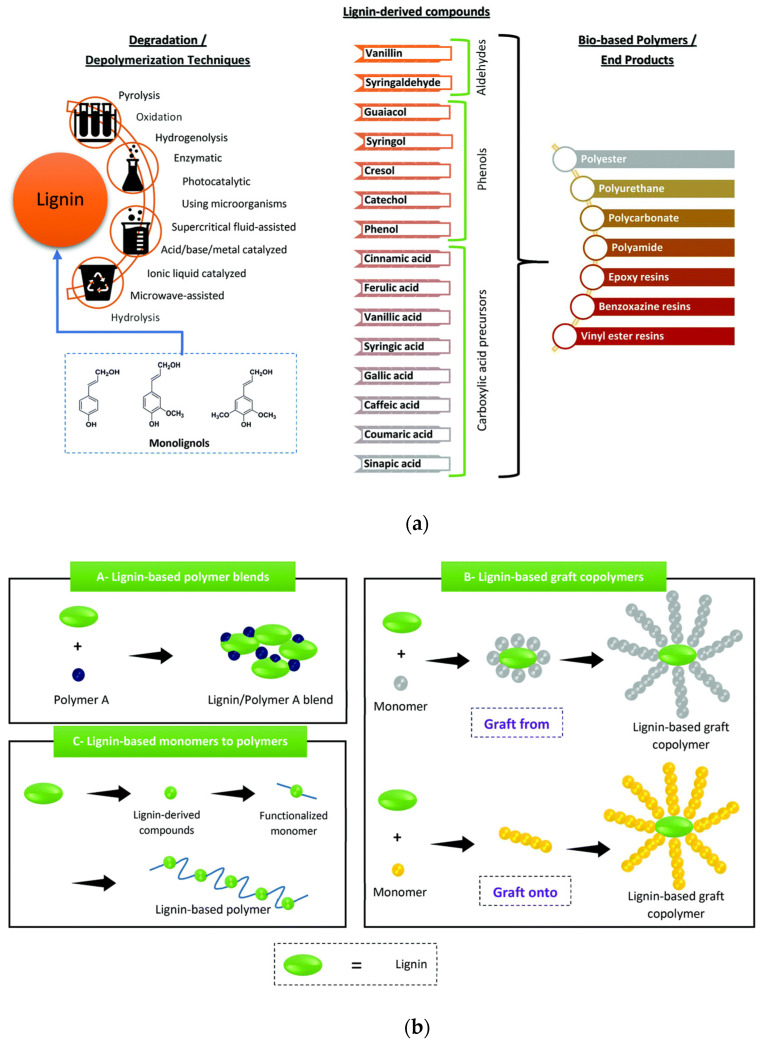
(**a**) Lignin depolymerization or degradation procedures and its derived monomers and polymers; (**b**) Different methods for lignin-based polymer synthesis. Reprinted with permission from Ref. [10]. Copyright 2021, Royal Society of Chemistry.

Different approaches can be considered to realize high-performance polymers from lignin. The different adopted depolymerization methods have great prospective to get several monomer precursors, e.g., as alcohols, aldehydes, and acids, from lignin (Figure 2a). Vanillin is one of the most explored precursors. Other derived lignin compounds are ferulic acid, coumaric acid, sinapic acid, vanillic acid, syringic acid, caffeic acid, cinnamic acid, syringaldehyde, guaiacol, syringol, phenol, cresol, and catechol. The different molecules can be arranged as blocks for various polymers (Figure 2b), e.g., polyesters, polycarbonates, phenolic resins, polyurethanes, and epoxy resins. Many reviews extensively refer to the synthesis of lignin-derived compounds and their use in the realization of bio-based polymers [11,12,13,14,15,16]: here, we will exclude the case of lignin polymer blends, while the possibility of graft polymerization or lignin conversion of monomers to polymers will be considered.

### 2.1. Synthesis Routes of Bio-Based Thermoplastics from Lignin-Based Molecules

**Figure 3:**
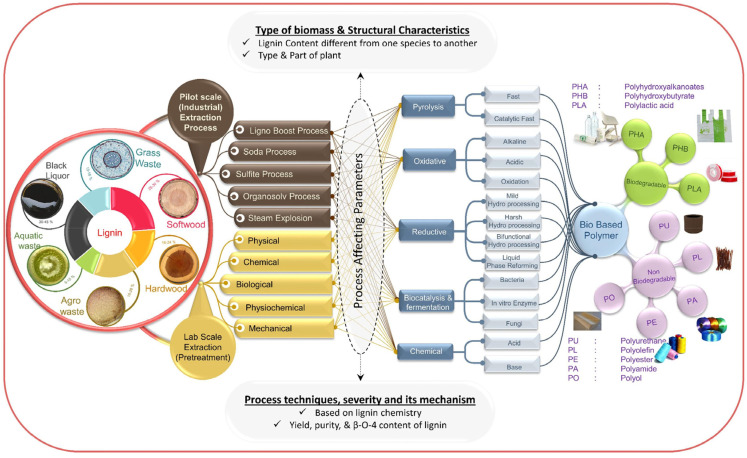
Schematic representation of lignin valorization on biopolymer production. Reprinted with permission from [17]. Copyright 2019, Elsevier Ltd.

The use of valorized lignin increases the potential to realize biodegradable biopolymers, such as polyhydroxyalkanoates, polyhydroxybutyrates, polylactic acid, or nonbiodegradable matrices, as in the case of polyurethane, polyolefins, polyamides, etc. [17] (Figure 3).

The first case of polymerized vanillic acid to make polyesters was defined in 1955 [18]. Carboxylate was obtained by the conversion of vanillic acid and by the esterification process of a phenolic segment with ethylene dihalides. After that, the carboxylate was chemically esterified with ethylene glycol and reduced to linear polyester, showing a glass transition temperature of 80 °C and a melting temperature of 210 °C [18]. In 1981, a similar strategy was established by Lange and coauthors to produce vanillic and syringic acid-based polymers. In the second path, the phenolic moiety of vanillic acid interacted with ethylene oxide [19]. In the refining technique, lignin has a complex structure that makes it hard to be directly adapted into high-value materials: nevertheless, with the advanced clarification of the lignin structure and its microbial metabolism, it has been possible to alter the lignin structure to give, for example, high-value-added products by means of biological procedures, as in the case of PHAs. In nature, numerous bacteria have settled various metabolic paths to convert lignin to PHAs with short-, medium-, or long-chain structures; specifically, lignin derivatives can be processed to acetyl-CoA for PHA. Currently, PHAs from lignin or lignin-related aromatic compounds have been obtained by selecting many bacteria, such as *Oceanimonas doudoroffii* (PHA from sinapinic acid and syringic acid), *Cupriavidus basilensis* B-8, *Pseudomonas putida* A514, and *Pseudomonas putida* KT2440. The produced PHA can be adapted to convert into varied chemical precursors, such as alkenoic acids and hydrocarbons, which indicate that lignin can be adapted to become fuel-range hydrocarbons, chemical precursors, and biomaterials [20].

Lignin has been also explored as a natural resource for the synthesis of polyurethanes because it retains hydroxyl groups on its surface [21]. It fits well even into PU chemistries, since it acts both as crosslinking agent, due to the accessibility of numerous hydroxyl groups on each molecule and as a hard segment due to the aromatic nature. The last years have also viewed a reliable tendency that seeks to take advantage of the vast availability of renewable feedstocks, such as lignin derivatives, terpenes, vegetable oils, and polyols as precursors for the synthesis of nonisocyanates polyurethanes (NIPUs). Many approaches have been explored to use the aforesaid renewable resources to synthetize NIPUs combined with the prerequisites of green chemistry [22].

**Figure 4:**
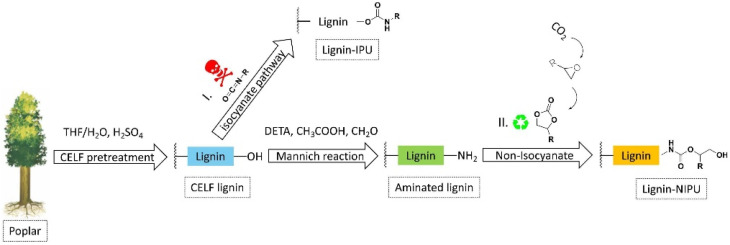
Synthesis procedures for polyurethanes obtained from lignin via (**I**) isocyanates pathway and (**II**) nonisocyanates way. Reprinted with permission from [23]. Copyright 2022, Elsevier Ltd.

Meng et al. studied a different method that utilizes lignin extracted from the cosolvent lignocellulosic fractionation (CELF) of poplar wood to realize bio-NIPUs [23]. In this method, hardwood poplar is initially reduced in different fractions via a CELF treatment with the aim of recovering a lignin stream rich in phenolic content. The CELF lignin was then aminated by a Mannich reaction [23] and finally reacted with bicyclic carbonates to produce an innovative NIPU. The authors suggested the utilization of the CELF reaction to obtain a lignin rich in phenolic OH groups to raise its reactivity in amination. In addition, the authors studied, via the Mannich reaction, the amination of CELF lignin by using diethylenetriamine and formaldehyde in acidic environments. Successively, as an alternative to the reaction of hydroxyl groups of lignin with isocyanate groups to gain the traditional PU linkage (pathway I, Figure 4), the amine group in the CELF lignin reacted with cyclic carbonate originated from carbonation of epoxides to give lignin-based NIPU (pathway II, Figure 4).

According to this, the mechanical behavior of NIPUs based on lignin can be definitely varied from rigid to elastic by basically changing the lignin constituents of the polymeric material. The thermal characteristics of NIPUs were enhanced thanks to the addition of aminated lignin, and NIPU containing 55 and 23 wt% of lignin exhibited a high elongation at break (~140%) and tensile strength (~1.2 MPa), respectively. The obtained experimental results reveal that the reaction of cyclic carbonate with aminated lignin can be considered as a significant strategy for the synthesis of lignin-based NIPU with a relatively high lignin amount [23]. Lignin can also work as a polyol in the polyester synthesis, and hydroxyl-based chemistries can be adapted to give terminal hydroxyl groups, carboxylic acid, acyl groups, and epoxy groups. Polyester copolymers based on lignin can be obtained by the reaction of hydroxyl groups with additional reactive functional groups (diacyl chloride, dicarboxylic acid, adipic acid, and/or phthalic anhydride): in the case of branched lignin-based poly (ester-amine), reactions of triethanolamine, lignin, and adipic acid have to be considered [24]. Copolymerization of vinyl monomers and lignin is usually applied to realize lignin-based vinyl polymeric systems by different radical initiation routes [25].

Graft copolymerization, or in general, derivatization reactions, can generate new lignin containing thermoplastics by incorporating technical lignin and synthetic material. By appropriately choosing low-glass-transition temperature chemistries, lignin derivatives with variable thermomechanical properties can be obtained. On the other hand, the new progress on controlled/living polymerization and specific and effective synthetic techniques (e.g., click chemistry) propose new pathways for the design and realization of high-performance thermoplastic materials based on lignin derivatives having functional properties [26]. Even with a fruitful commercialization of many lignin-containing thermoplastics, constant efforts are still required to advance and create a new generation of lignin-based thermoplastics with precise structures, strong melt workability, good mechanical, and thermal performances.

### 2.2. Synthesis Routes of Bio-Based Thermosets from Lignin-Based Molecules

Thermosetting polymers can be produced from many bio-based resources, as in the case of vegetable oils, lactic acid, and citric acid, giving polymeric materials with more than 90% of a renewable amount [27]. Various bio-based thermosetting resins have been considered through the manipulation of virgin renewable feedstock; therefore, research on how to properly convert residual biomass to stimulate the production of new materials with remarkable properties is of great impact. Many functionalization approaches, including chemical or physical modifications, have been taken into account to broaden the application fields of these new resins with a singular glance to their processability and recyclability [28,29]. Referring to lignin, its aromatic structure gives molecular rigidity, and providing high-glass-transition temperatures and stiffness, as well the presence of aliphatic and aromatic hydroxyl functionalities, is a crucial characteristic for application where high thermal stability and high network levels are required [30]. Nevertheless, employing lignin as a raw material is still a noteworthy duty, being characteristically heterogeneous in its native form. Moreover, the processes considered to obtain lignin from biomass permanently alters the assembly of the lignin backbone, which is cleaved, fractionated, and assembled, making it practically nonidentifiable from its source in nature. Additionally, new functional groups are inserted through, for example, oxidation reactions, forming carboxylic acids, aldehydes, and ketones [31]. Oxypropylation, allylation, epoxidation, acetylation, and silylation are a few of the pathways for the modification of technical lignin found in the literature, which makes it compliant to be incorporated, by compatibilization, in polymeric matrices.

**Figure 5:**
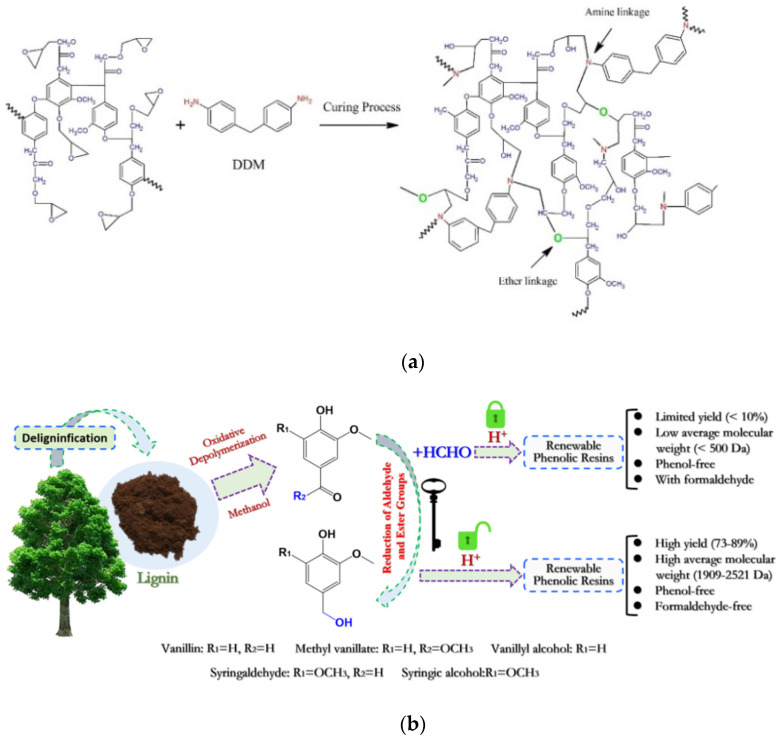
(**a**) Schematic representation and chemical structure of lignin-based epoxy resin and (**b**) schematic representation of the renewable phenolic resin synthesis based on lignin-derived monomers. Reprinted with permission from [34,35]. Copyright 2021, Elsevier Ltd.

Two approaches are currently considered to obtain lignin-based thermosets. The first method utilizes lignin itself or incorporates lignin with other components to synthesize copolymers, with the key restriction that the reactivity of bulk lignin is lower than that of monomers. To overcome these restrictions, another method was developed that uses aromatic molecules obtained directly from lignin depolymerization. This approach enables molecular design and structural modification and can increase the performance of the resulting polymeric materials, as in the case, for example, of vanillin and other derivatives [32]. The main task in both cases is still to obtain reproducible and well-characterized fractions. These prospective polymer feedstocks, on the other hand, have their own limited challenges in terms of yields, prepolymerization reactions, and workability. The review from Feghali et al. [14] presents an overview on polymers obtained from lignin-based model compounds and depolymerized lignin (vanillin, vanillic acid, aromatic acid, quinones, and aromatic aldehydes by oxidation reaction): an extensive multiplicity of high-performance polymeric systems, such as polyurethane (PU), epoxy resin, phenol formaldehyde, and polyester, exhibiting good thermal and mechanical characteristics, can be synthesized with lignin as the macromonomer [33]. For example, Fersosian [34] obtained high-yield phenolic monomers through the selective cleaving of the β-O-4 bond of native lignin for the synthesis of lignin-based epoxy resin; however, the severe depolymerization condition, the low monomer yields, and the high separation costs limit the industrial use of this strategy (Figure 5a).

Compatibility and reactivity of lignin with compounds can be enhanced by means of chemical changes (e.g., propoxylation, phenolation, demethylation, and esterification reactions). Nevertheless, steric-hindrance influence and the limited compatibility of lignin-based epoxy resin weakened the crosslinking density, deteriorating the thermomechanical behavior of the thermosetting polymeric materials. Consequently, limitations such as the complex process, limited effect, low-lignin loading, and waste liquid recovery are unavoidable and still need to be solved. In lignin-modified phenolic resins, lignin is considered as the phenol able to react with formaldehyde in basic conditions or as an aldehyde to react with phenol in acidic conditions. Nevertheless, the replaced amount of phenol is partial due to scarce reactive sites and steric hindrance in lignin. To overcome these restrictions, lignin-derived phenols have been exploited to produce phenolic resins [35] (Figure 5b). The production of renewable, green, and sustainable phenolic resins based on lignin-derived monomers, having the potential to substitute traditional polymers, is currently under intensive study.

## 3. Nanolignin as Filler in Polymeric Nanocomposites

Lignin nanoparticles have received much attention in the last years concerning the effort to utilize and apply lignin into more valued sectors [36]. In order to progress in the suitable use of lignin into different fields, it is required to ensue with chemical changes, fractionations to produce homogeneous materials, as previously described, or realize precipitated material with submicron particles for easier dispersion and enhanced features. The academic interest moved to the preparation of lignin nanoparticles (LNPs) by discovering their potential uses [37,38]. To date, the studied routes for the synthesis of LNPs are essentially chemical-based procedures which include, but are not limited to, acid-catalyzed, flash and nanoprecipitation, dialysis, solvent exchange, antisolvent process, W/O microemulsion processes, homogenization, and sonochemical synthesis. These methods have their profits and restrictions when they are utilized for LNP extractions. Therefore, the synthesis should be selected proficiently in order to yield LNPs of chosen sizes and dimensions [37,39]. Different methods, such as freeze-drying and thermal stabilization, interfacial crosslinking, polymerization and emulsion, and microbial- and enzyme-mediated, have been also considered as appropriate for the production of lignin nanoparticles. A comprehensive list of procedures that can be implemented has been reviewed in a few recent papers [40,41,42,43]. The procedures may give rise to appropriate advantages, but even distinctive faults regarding industrial use, since in some cases huge contents of solvents are necessary for the purification before precipitation, precipitation itself, and downstream processing, and in other cases a limited scalability of nanolignin production steps is manifest. Regardless the production yield, the research of suitable combination of LNPs with green matrices has progressed and prospective applications have been found and developed due to the specific characteristic of this material that can be considered for numerous potential applications (high thermal stability, manifest antioxidant properties, biodegradability, and UV-absorption features).

### 3.1. Nanolignin as Functional Filler in Thermoplastic Green Nanocomposites: Properties and Applications

**Figure 6:**
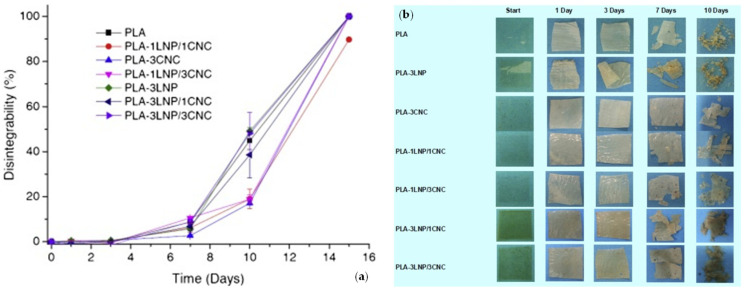
Disintegrability values (**a**) and visual images (**b**) of PLA and PLA binary and ternary nanocomposites at different incubation times in composting conditions. Reprinted with permission from Ref. [46]. Copyright 2016, Elsevier Ltd.

The use of nanolignin as a reinforcing phase in macromolecules (both natural and synthetic) is a key methodology to advance in the realization of sustainable polymeric composite systems. Recently, lignin was utilized as a nanoscaled reinforcement to improve the structural characteristics of polysaccharides, proteins, natural rubber, and synthetic polymeric matrices [44,45]. In this context, the advance in the modification of lignin-based materials to give nanocomposites is, in the last decades, evident, due to the growing interest of the academic and industrial area. To provide few examples, Yang and coauthors proposed the use of lignin nanoparticles (LNP) in poly (lactic acid). They selected two different amounts (1 and 3% wt.) of nanofillers to be utilized in the polymer. Data obtained from antimicrobial analysis demonstrate the ability to hinder the growth of *Xanthomonas axonopodis pv*. *vesicatoria* and *Xanthomonas arboricola pv. pruni* Gram-negative bacteria over time, to positively influence the innovation, and to induce a positive effect against hazardous bacterial plant pathogens. The disintegration test under composting conditions revealed that the tested formulations reach a value up to 90% after 15 days; however, the presence of LNPs did not affect the disintegrability of different films, as shown in Figure 6a,b. The presence of LNPs did not affect the migration value, and accordingly the polymeric systems can be regarded as appropriate for the food packaging sector [46]. Data obtained from antimicrobial analysis demonstrate the ability to hinder the growth of *Xanthomonas axonopodis pv. vesicatoria* and *Xanthomonas arboricola pv. pruni* Gram-negative bacteria over time, to positively influence the innovation, and to induce a positive effect against dangerous bacterial plant pathogens. The disintegration test under composting conditions revealed that the tested formulations reach a value up to 90% after 15 days; however, the presence of LNPs did not affect the disintegrability of different films, as shown in Figure 6a,b. The presence of LNPs did not affect the migration value, and accordingly the films can be regarded as appropriate for application in the food packaging sector [46].

Chollet and coauthors considered the nanolignin as a new additive for flame-retardancy of poly (lactic acid) [47]. Lignin nanoparticles (LNPs) have been obtained from Kraft lignin microparticles by considering a dissolution–precipitation process. Micro- and nanolignins chemistries were altered by functionalizing the external surface with diethyl chlorophosphate (LMP-diEtP and LNP-diEtP, respectively) and diethyl (2-(triethoxysilyl)ethyl) phosphonate (LMP-SiP and LNP-SiP, respectively) to improve their flame-retardant effect in PLA. The results of inductively coupled plasma (ICP) spectrometry demonstrated that a great content of phosphorus was grafted onto the nanoparticles. Nevertheless, phosphorylated lignin nanoparticles limited PLA degradation during melt processing and the nanocomposite systems were shown to be relatively stable from the thermal point of view.

**Figure 7:**
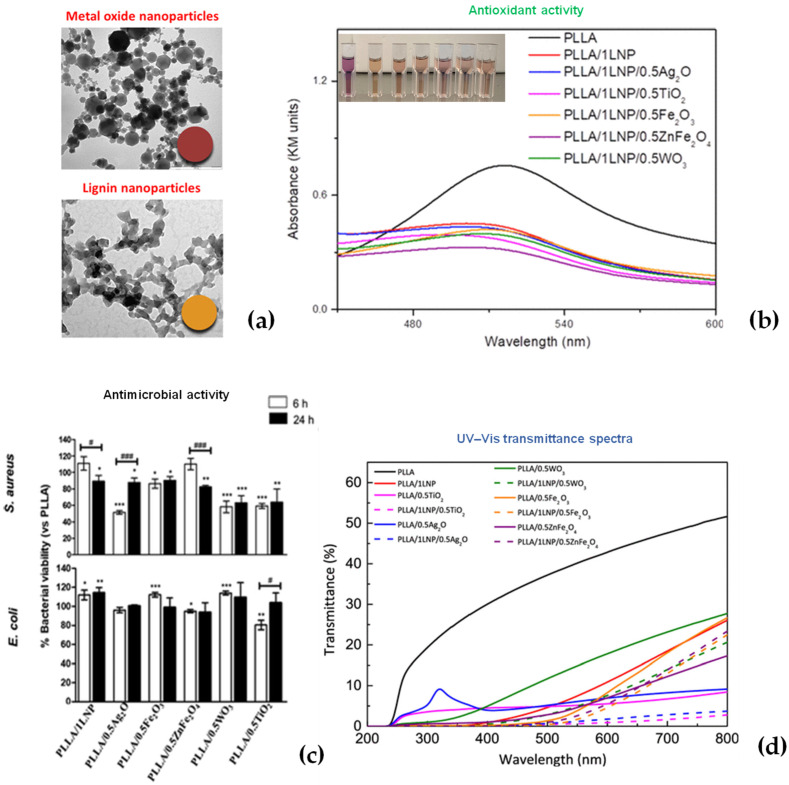
(**a**) TEM images of metal oxide (Fe_2_O_3_) and lignin nanoparticles (LNPs); (**b**) antioxidant activities of PLLA-based systems, antimicrobial activities; (**c**) antimicrobial activities of PLLA binary systems; (**d**) UV–vis spectra of PLLA binary and ternary films. Reprinted from [49]. Copyright 2020 American Chemical Society.

The use of lignin nanoparticles was largely applied and investigated as a method to develop new multifunctional, innovative materials in the food packaging sector. LNPs have been confirmed to provide enhanced mechanical, thermal, and antioxidant characteristics to the polymers in which they are incorporated depending on their particle size [48]). Lizundia et al. proposed the development of poly(l-lactide) (PLLA)-based nanosystems realized by the solvent casting method and combining LNPs with various metal oxide nanoparticles, such as WO_3_, Ag_2_O, Fe_2_O_3_, TiO_2_, and ZnFe_2_O_4_ [49] (Figure 7a). It was found that the formulations based on nanolignin and ZnFe_2_O_4_ particles exhibited the best antioxidant behavior. Radical scavenging activity was also observed in ternary-based nanocomposites, where lignin and metal oxide nanofillers operated together synergically to boost the functional properties (Figure 7b). The antimicrobial activity of binary nanocomposites containing metal oxide NPs was correspondingly strong against PLLA, but it was only persistent for a few ternary nanocomposite films in a time result that was more obvious for *S. aureus* than for *E. coli* (Figure 7c). Lignin nanoparticles can protect towards UV light while allowing visible light to get through, and they can exceed the UV protection effect of numerous inorganic nanoparticles (Figure 7d).

The central role of lignin nanoparticles as UV barrier filler was also investigated by Yang and coauthors [50]. They combined LNPs in PLA and polycaprolactone (PCL)-based formulations to increase packaging ductility and UV barrier properties. LNPs and caprolactone were first diluted in toluene to obtain a homogeneous solution and then purged with N_2_ gas. The process was maintained at 120 °C for two days after the l-lactide addition. The addition of PCL determined an increase of the elongation at break up to 185%, an initial decrease of tensile strength that gradually increased to 280% after the addition of the LNP-P(LA-CL) copolymer. The toughness also rose 1.5 times above the PLA/PCL. Similar results were observed for the crystallization values and UV protection. Nanolignin–PLA/PCL-based systems can be utilized in the food packaging industry as an impact-resistant and UV-protectant material.

Cavallo et al. suggested the use of polylactic acid (PLA) films containing 1 wt% and 3 wt% of lignin nanoparticles (pristine (LNP) chemically modified with citric acid (caLNP) and acetylated (aLNP)). The different polymeric films were produced by extrusion and filming, and after that, the formulations were analyzed by determining the overall performance needed for the food packaging sector [51]. The obtained data indicated that all lignin nanoparticles induced UV-blocking, and antioxidant and antibacterial (against Gram-positive *Micrococcus luteus* and Gram-negative *Escherichia coli* bacteria) behavior to the PLA films, and a higher consequence was indeed found when increasing the filler content. Acetylation (aLNP) of the fillers moderately limited the antioxidant characteristics and the UV protection of the obtained composite systems, but it affected positively the nanoparticles distribution and aggregation, improving ductility and aesthetic quality of the films by decreasing at the same time the characteristic dark color of the lignin. Migration tests and disintegration test realized in simulated composting conditions of the nanocomposites showed that, irrespectively of their system, the realized active nanocomposites behaved likewise to neat PLA.

**Figure 8:**
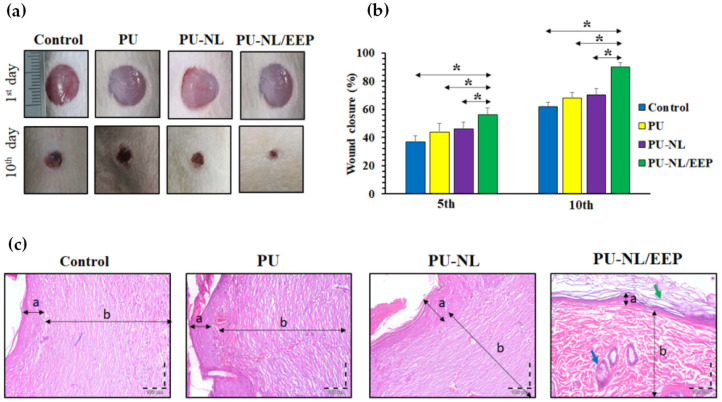
(**a**) Visual images of the wounds after 1 and 10 days of postoperation for the PU, PU-LNP, and PU-LNP/EEP groups. (**b**) Histograms of the wound closure for PU, PU-LNP, and PU-LNP/EEP groups. The data are expressed as mean ± standard deviation, (n = 8, *: *p* < 0.05, ns: not significant). (**c**) H&E-stained sections of skin specimens from the wound site of PU, PU-LNP, and PU-LNP/EEP groups. Arrows (a and b letters) designate the epidermis and dermis layers, respectively. Green and blue arrows indicate the keratin layer and sebaceous gland, respectively. Reprinted from Ref. [52]. Copyright 2021 MDPI AG.

The use of LNPs was proposed as a valid possibility to develop promising wound dressing. Pahlevanneshan and coauthors [52] proposed the design and characterization of porous nanocomposite based on polyurethane (PU) foam synthesis. Moreover, the developed materials containing nanolignin coated with natural antimicrobial propolis for wound dressing. The antimicrobial effect was observed adding the extract to the polymeric foams, and all foams showed high biocompatibility toward L929 fibroblast cells, with the highest cell viability and cell attachment in the case of PU-LNP/propolis extract. In vivo wound-healing results, obtained by using Wistar rats’ full-thickness skin wound model, showed that PU-LN/EEP has advanced wound-healing efficiency when compared to foams (Figure 8a–c) [52].

### 3.2. Nanolignin as Functional Filler in Thermosetting Green Nanocomposites: Properties and Applications

**Figure 9:**
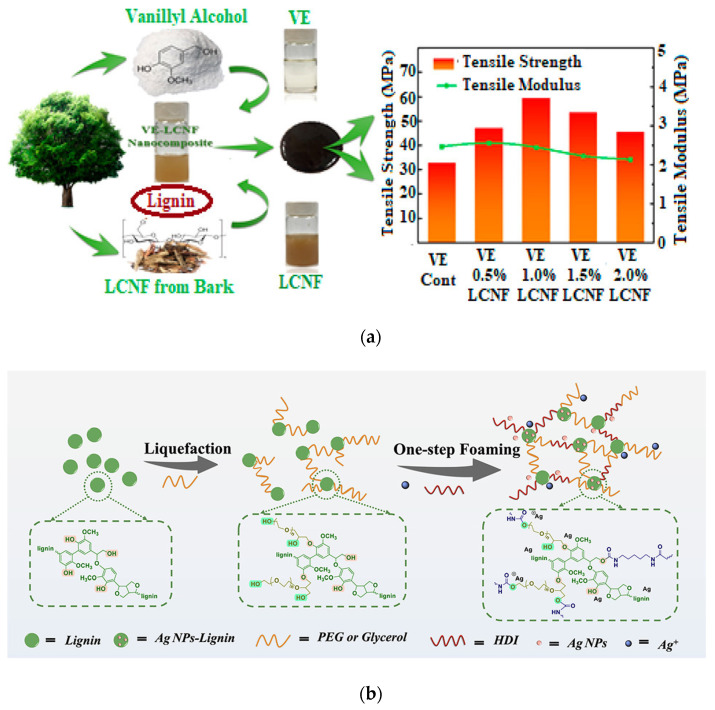
(**a**) Vanillin-based epoxy resin (VE) reinforced with lignin-containing cellulose nanofibrils (LCNFs) and the results of mechanical performance of the nanocomposites produced by considering different LCNF contents; (**b**) Schematic drawing of Ag NP-LPUF composite foam preparation by lignin liquefaction and one-step foaming. Reproduced with permission from [56,58]. Copyright 2020 and Copyright 2022, American Chemical Society.

Lignin can be blended, considered as a filler in a composite/nanocomposite formulation, both in its native form or chemically modified, combined in the presence of particular additives: in all cases, it has been proved that lignin can beneficially improve the overall performance of the resulting polymers. While the literature reports numerous examples of LNPs in thermosets [53,54,55], limited cases of nanolignin incorporation in green-based thermosetting matrices can be found. In their paper, Wang et al. [56] considered a simple and fast synthesis method to synthetize bio-based epoxy resin obtained from vanillyl alcohol; after that, vanillin-based epoxy resin (VE) was additionally reinforced by lignin-containing cellulose nanofibrils (LCNFs) with different weight contents. The authors experimentally observed that a significant improvement in the thermomechanical performance of the nanocomposites was attained with a low amount of nanofibril addition, confirming the possibility of assembling environmentally friendly and sustainable bio-based epoxy lignin nanocomposites with superior properties (Figure 9a).

A new approach was adopted to realize lignin phenol formaldehyde (LPF) resin: in the paper of Chen et al. [57], the preparation of nanolignin with a high specific surface area and porous structure was arranged, and this nanofiller was then utilized as a valid phenol substitute combined with formaldehyde to produce a wood adhesive. Data showed that replacement of phenol by nanolignin could enhance the thermal characteristic of the resin, and in parallel, the modification of the curing schedule of the prepared lignin-based resin was considered.

In a quite recent paper [58], a simple foaming process to realize lignin-based polyurethane foams (LPUFs) was also considered: in that specific case, bio-based polyether polyols partially replaced petroleum-based raw components. Traces of phenolic hydroxyl groups (about 4 mmol) in lignin functioned as a direct reducing component and capping agent to silver ions by forming in situ silver nanoparticles (Ag NPs) within the LPUF skeleton. The lignin polyurethane/Ag composite foam (named as Ag NP-LPUF) was characterized by modulated thermomechanical and antibacterial properties, confirming the possibility of using these antimicrobial composite foams to encourage wound healing of full-thickness skin defects (Figure 9b).

Another example of lignin nanoparticle exploitation in a novel manner is represented by the study reported by [59], where the authors considered the realization of water-based, solvent-free, and multiresistant surface coatings: due to the presence of hydroxyl groups, the nanolignin acted as a hardener and no binder was required to realize adhesion to the substrate. In the case of the wood substrate, the particle morphology permitted proficient water repellency with a low coating weight, since the coating maintained the surface roughness of the wooden substrate while providing additional hydrophobicity.

Researchers from Washington State University, part of the NSF-supported Industry–University Cooperative Research Center for Bioplastics and Biocomposites (CB2), considered the use of a deep eutectic solvent to extract oligomeric lignin (nanoDESL) from plant biomass at a high yield and also nanosized [60]. NanoDESL shows narrower molecular size dimensions, distribution, and structural characteristics of traditional lignin. Oxypropylation of lignin was also optimized: it has been revealed that the use of polar aprotic solvents for the oxypropylation coupled with nanoDESL significantly promotes the oxypropylation reaction toward the synthesis of semiflexible PU. It was observed that the lignin-based PU containing ~20 wt% nanoDESL realized using polyol had density and compressive force comparable to the “standard” PU foam. The researchers are investigating how to enhance the reaction yield, with the goal of including 40 wt% lignin-based polyol into semiflexible foams: the potential of nanoDESL-based PU for adhesive, sealant, and coating applications is also explored by also considering their environmental toxicity and biodegradability issues. Using lignin as a source for PU synthesis not only encourages a circular economy but may also lead to the design of more ecofriendly end-of-life routes for PU plastics.

All these studies provide awareness on potentialities of lignin nanosized fractions and allows for the design of reproducible and foreseeable material characteristics. It is essential to know these characteristics if we would exploit lignin as a raw material for a sustainable and innovative design. These preliminary and updated works confirm that nanolignin, if combined with green thermosetting matrices, can give fully green nanocomposites and, if effective, multifunctionality is often achieved in the presence of this nanoscaled filler.

## 4. Conclusions and Future Perspectives

This review, divided into two main sections, firstly provided an overview of preparation and applications of green thermoplastic and thermosetting nanocomposites based on lignin, and thereby a glance to the use of nanolignin in biopolymeric nanocomposites was also considered. Even if various structures and different properties of lignin at the nanoscale effectively can be challenging and interesting from the research point of view, the preparation and application of nanolignin-based green composites in high-value sectors are still in their infancy. Limiting factors include the achievement of uniform dispersion, and, additionally, the morphology, size, and chemistry of lignin nanoparticles, which need to be the prerequisites for the high-value-added and multifield applications of lignin. The structural and functional properties of lignin are the key points for its conversion into aromatics, polymers, and high-performance materials. Regarding this issue, we should emphasize that the production of thermoplastics or thermosetting polymers from the depolymerized lignin still involves the use of several chemicals, often causing a high environmental impact for the synthesis of bio-based and/or biodegradable polymers. Consequently, methods to chemically functionalize lignin to useful products without the use of expensive reagents or complicated synthetic routes must still be identified, with the main aim of competing with commercial commodity polymeric materials. The success of synthesizing thermoplastics and thermosetting from lignin opens up, on the other hand, new avenues to incorporate lignin as a component of value-added polymers while utilizing renewable resources. To balance the negative impact of chemically treated lignin and lignin derivatives to produce bio-based matrices, the use of lignin nanoparticles (obtained applying green process) in lignin-derived polymeric nanocomposites can be considered as a valid strategy to guarantee multifunctionality in sustainable biopolymeric matrices.

## Data Availability

Not applicable.
